# Supplementary material for: The causal relationship between the human gut microbiota and pyogenic arthritis: a Mendelian randomization study
Source: Front Cell Infect Microbiol. 2024 Nov 26;14:1452480. doi: 10.3389/fcimb.2024.1452480 (PMC11629706; doi:10.3389/fcimb.2024.1452480)
Supplement: Supplementary file 1 [file DataSheet1.zip › mendelian test/ebi-a-GCST90027575.csv_leave_one_out.pdf]

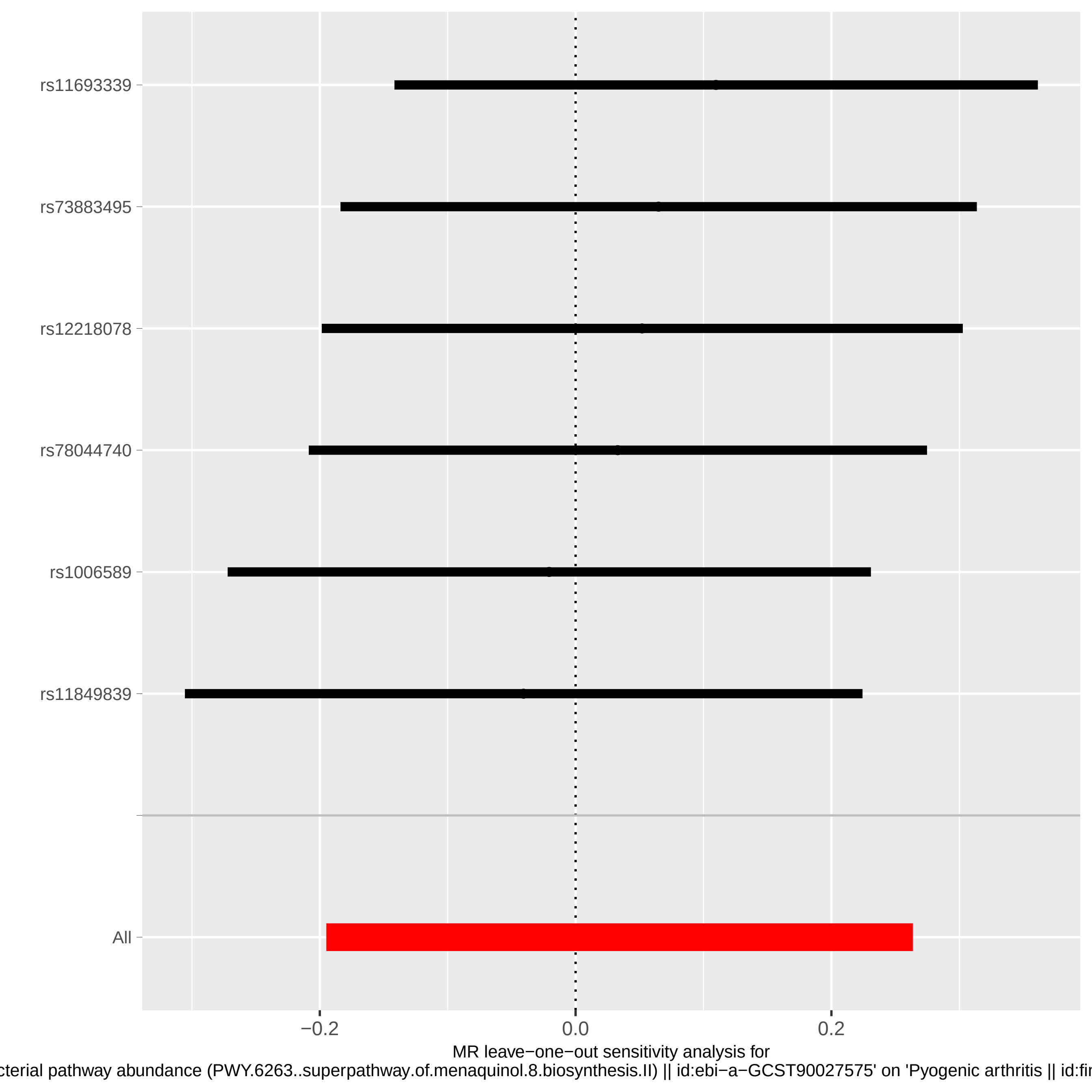
rs11693339
rs73883495
rs12218078
rs78044740
rs1006589
rs11849839
All
−0.2
0.0
0.2
MR leave−one−out sensitivity analysis for
cterial pathway abundance (PWY.6263..superpathway.of.menaquinol.8.biosynthesis.II) || id:ebi−a−GCST90027575' on 'Pyogenic arthritis || id:fir
